# Supplementary material for: Diversity and Evolution of Viral Pathogen Community in Cave Nectar Bats (Eonycteris spelaea)
Source: Viruses. 2019 Mar 12;11(3):250. doi: 10.3390/v11030250 (PMC6466414; doi:10.3390/v11030250)

Adenovirus (V4)

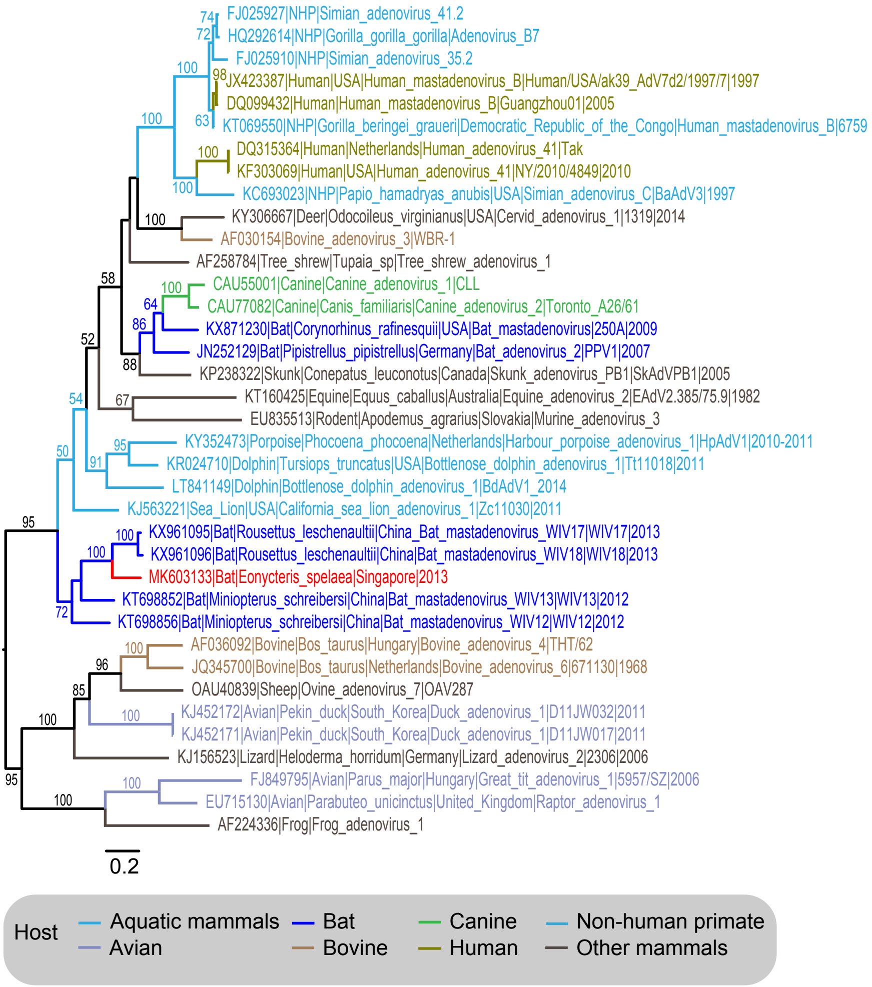

Supplement: Supplementary file 1 [file viruses-11-00250-s001.zip › 2-viruses-447243-suppl/SFig_01_Adenovirus.pdf]
