# Supplementary material for: Diversity and Evolution of Viral Pathogen Community in Cave Nectar Bats (Eonycteris spelaea)
Source: Viruses. 2019 Mar 12;11(3):250. doi: 10.3390/v11030250 (PMC6466414; doi:10.3390/v11030250)
Supplement: Supplementary file 1 [file viruses-11-00250-s001.zip › 2-viruses-447243-suppl/SFig_04_Picornavirus.pdf]

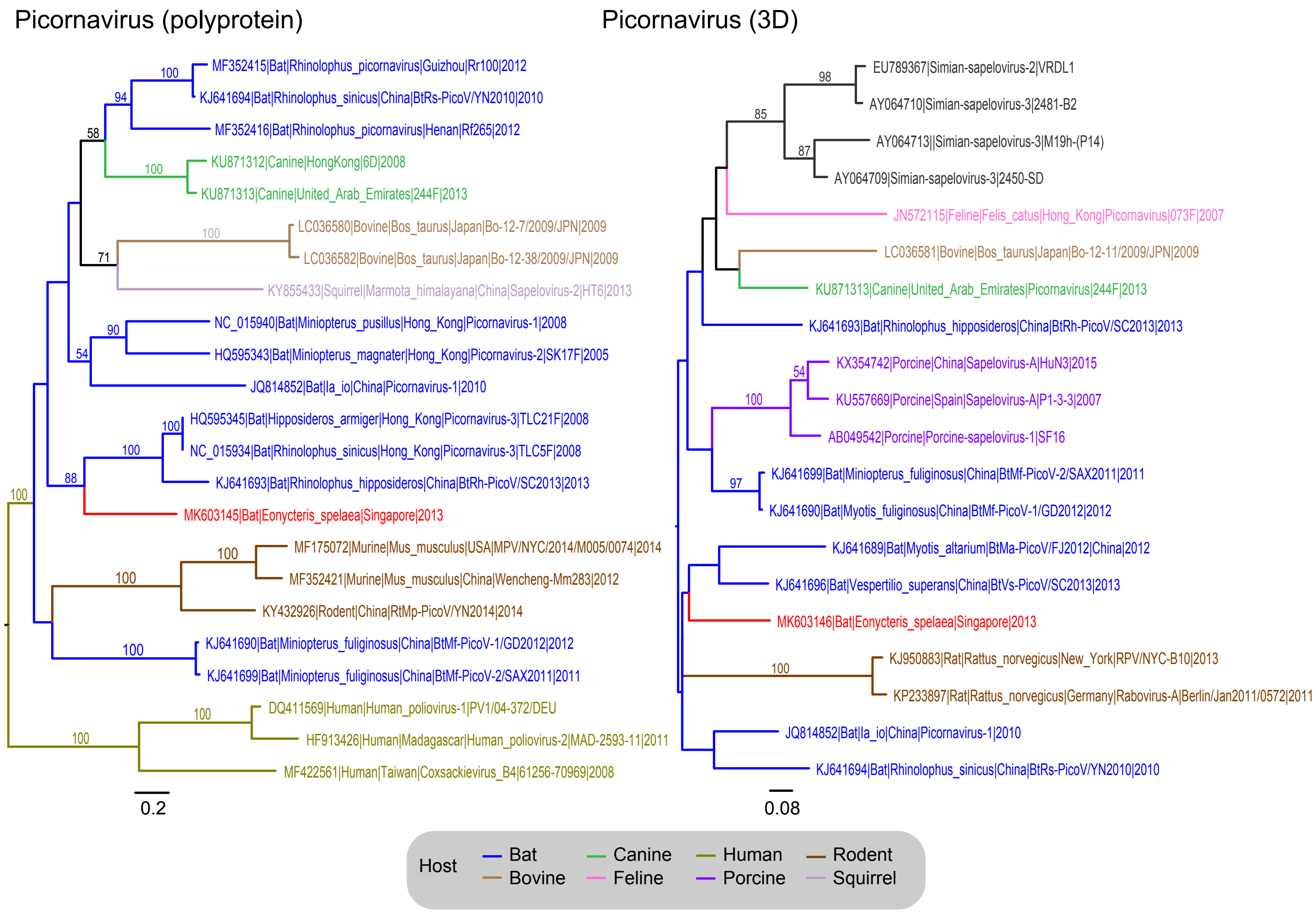

Picornavirus (polyprotein)
MF352415|Bat|Rhinolophus_picornavirus|Guizhou|Rr100|2012
KJ641694|Bat|Rhinolophus_sinicus|China|BtRs-PicoV/YN2010|2010
MF352416|Bat|Rhinolophus_picornavirus|Henan|Rf265|2012
KU871312|Canine|HongKong|6D|2008
KU871313|Canine|United_Arab_Emirates|244F|2013
LC036580|Bovine|Bos_taurus|Japan|Bo-12-7/2009/JPN|2009
LC036582|Bovine|Bos_taurus|Japan|Bo-12-38/2009/JPN|2009
KY855433|Squirrel|Marmota_himalayana|China|Sapelovirus-2|HT6|2013
NC_015940|Bat|Miniopterus_pusillus|Hong_Kong|Picornavirus-1|2008
HQ595343|Bat|Miniopterus_magnater|Hong_Kong|Picornavirus-2|SK17F|2005
JQ814852|Bat|Ia_io|China|Picornavirus-1|2010
HQ595345|Bat|Hipposideros_armiger|Hong_Kong|Picornavirus-3|TLC21F|2008
NC_015934|Bat|Rhinolophus_sinicus|Hong_Kong|Picornavirus-3|TLC5F|2008
KJ641693|Bat|Rhinolophus_hipposideros|China|BtRh-PicoV/SC2013|2013
MK603145|Bat|Eonycteris_spelaea|Singapore|2013
MF175072|Murine|Mus_musculus|USA|MPV/NYC/2014/M005/0074|2014
MF352421|Murine|Mus_musculus|China|Wencheng-Mm283|2012
KY432926|Rodent|China|RtMp-PicoV/YN2014|2014
KJ641690|Bat|Miniopterus_fuliginosus|China|BtMf-PicoV-1/GD2012|2012
KJ641699|Bat|Miniopterus_fuliginosus|China|BtMf-PicoV-2/SAX2011|2011
DQ411569|Human|Human_poliovirus-1|PV1/04-372/DEU
HF913426|Human|Madagascar|Human_poliovirus-2|MAD-2593-11|2011
MF422561|Human|Taiwan|Coxsackievirus_B4|61256-70969|2008
100
94
58
100
100
71
90
54
100
100
88
100
100
100
100
100
100
0.2
Picornavirus (3D)
EU789367|Simian-sapelovirus-2|VRDL1
AY064710|Simian-sapelovirus-3|2481-B2
AY064713||Simian-sapelovirus-3|M19h-(P14)
AY064709|Simian-sapelovirus-3|2450-SD
JN572115|Feline|Felis_catus|Hong_Kong|Picornavirus|073F|2007
LC036581|Bovine|Bos_taurus|Japan|Bo-12-11/2009/JPN|2009
KU871313|Canine|United_Arab_Emirates|Picornavirus|244F|2013
KJ641693|Bat|Rhinolophus_hipposideros|China|BtRh-PicoV/SC2013|2013
KX354742|Porcine|China|Sapelovirus-A|HuN3|2015
KU557669|Porcine|Spain|Sapelovirus-A|P1-3-3|2007
AB049542|Porcine|Porcine-sapelovirus-1|SF16
KJ641699|Bat|Miniopterus_fuliginosus|China|BtMf-PicoV-2/SAX2011|2011
KJ641690|Bat|Myotis_fuliginosus|China|BtMf-PicoV-1/GD2012|2012
KJ641689|Bat|Myotis_altarium|BtMa-PicoV/FJ2012|China|2012
KJ641696|Bat|Vespertilio_superans|China|BtVs-PicoV/SC2013|2013
MK603146|Bat|Eonycteris_spelaea|Singapore|2013
KJ950883|Rat|Rattus_norvegicus|New_York|RPV/NYC-B10|2013
KP233897|Rat|Rattus_norvegicus|Germany|Rabovirus-A|Berlin/Jan2011/0572|2011
JQ814852|Bat|Ia_io|China|Picornavirus-1|2010
KJ641694|Bat|Rhinolophus_sinicus|China|BtRs-PicoV/YN2010|2010
98
85
87
54
100
97
100
0.08
Host
Bat
Bovine
Canine
Feline
Human
Porcine
Rodent
Squirrel
