# Supplementary material for: Diversity and Evolution of Viral Pathogen Community in Cave Nectar Bats (Eonycteris spelaea)
Source: Viruses. 2019 Mar 12;11(3):250. doi: 10.3390/v11030250 (PMC6466414; doi:10.3390/v11030250)
Supplement: Supplementary file 1 [file viruses-11-00250-s001.zip › 2-viruses-447243-suppl/viruses-447243-supplementary captions.pdf]

Supplementary Figures captions:

Supplementary Figure 1. Phylogenetic relationships of the V4 gene sequences of adenovirus, inferred by using the maximum-likelihood method with the GTR+GAMMA model in RAxML. Coloured branches represent viruses isolated from different hosts. Red branches denote new sequences collected from *Eonycteris spelaea* bats in Singapore. Bootstrap support values greater than 50% are displayed at major nodes. The scale bar indicates the number of nucleotide substitutions per site.

Supplementary Figure 2. Phylogenetic relationships of the E and NS5 gene sequences of flavivirus, inferred by using the maximum-likelihood method with the GTR+GAMMA model in RAxML. Coloured branches represent viruses isolated from different hosts. Red branches denote new sequences collected from *Eonycteris spelaea* bats in Singapore. Bootstrap support values greater than 50% are displayed at major nodes. The scale bar indicates the number of nucleotide substitutions per site.

Supplementary Figure 3. Phylogenetic relationships of the E1 gene sequences of papillomavirus, inferred by using the maximum-likelihood method with the GTR+GAMMA model in RAxML. Coloured branches represent viruses isolated from different hosts. Red branches denote new sequences collected from *Eonycteris spelaea* bats in Singapore. Bootstrap support values greater than 50% are displayed at major nodes. The scale bar indicates the number of nucleotide substitutions per site.

Supplementary Figure 4. Phylogenetic relationships of the polyprotein and 3D gene sequences of picomavirus, inferred by using the maximum-likelihood method with the GTR+GAMMA model in RAxML. Coloured branches represent viruses isolated from different hosts. Red branches denote new sequences collected from *Eonycteris spelaea* bats in Singapore. Bootstrap support values greater than 50% are displayed at major nodes. The scale bar indicates the number of nucleotide substitutions per site.

Supplementary Figure 5. Phylogenetic relationships of the VP2 sequences of polyomavirus, inferred by using the maximum-likelihood method with the GTR+GAMMA model in RAxML. Coloured branches represent viruses isolated from different hosts. Red branches denote new sequences collected from *Eonycteris spelaea* bats in Singapore. Bootstrap support values greater than 50% are displayed at major nodes. The scale bar indicates the number of nucleotide substitutions per site.
