# Supplementary figures and images for: Diversity and Evolution of Viral Pathogen Community in Cave Nectar Bats (Eonycteris spelaea)
Source: Viruses. 2019 Mar 12;11(3):250. doi: 10.3390/v11030250 (PMC6466414; doi:10.3390/v11030250)

## Flavivirus (E)

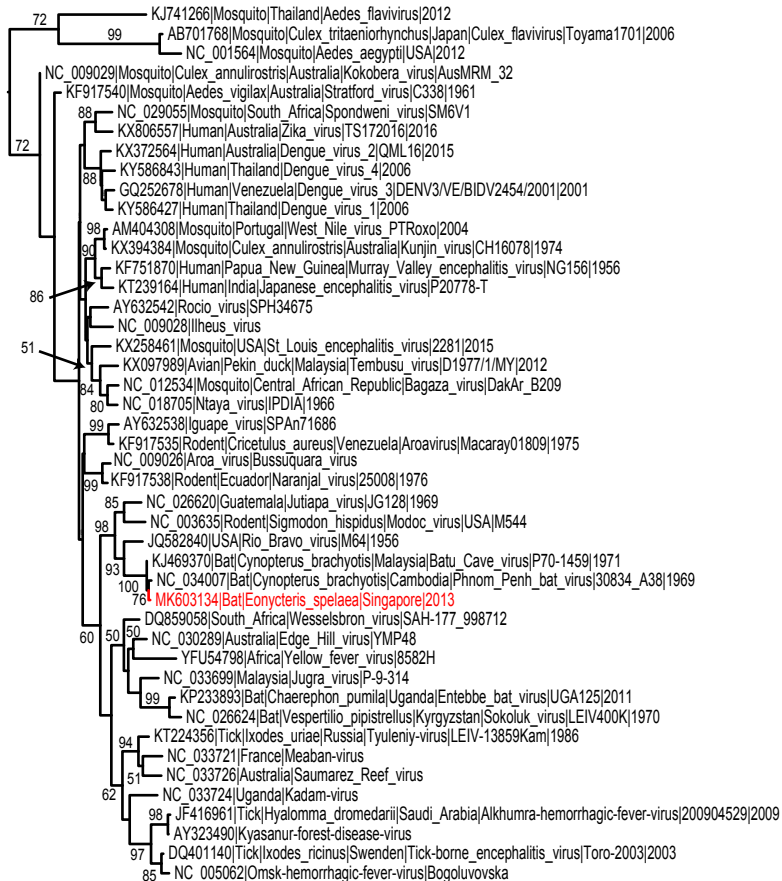

0.5

## Flavivirus (NS5)

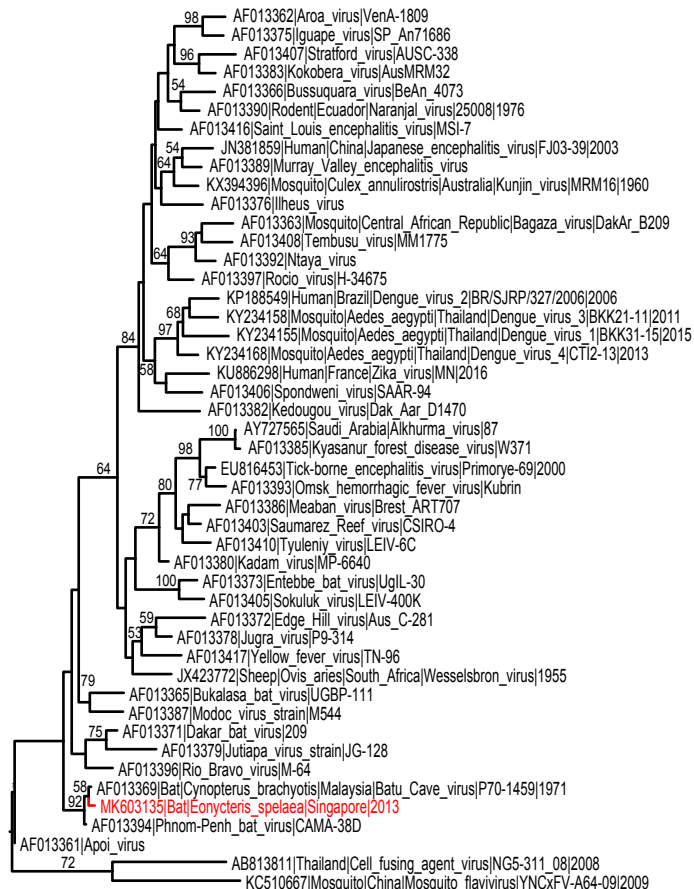

0.3

Supplement: Supplementary file 1 [file viruses-11-00250-s001.zip › 2-viruses-447243-suppl/SFig_02_Flavivirus_revised_Mar1.pdf]

# Papillomavirus (E1)

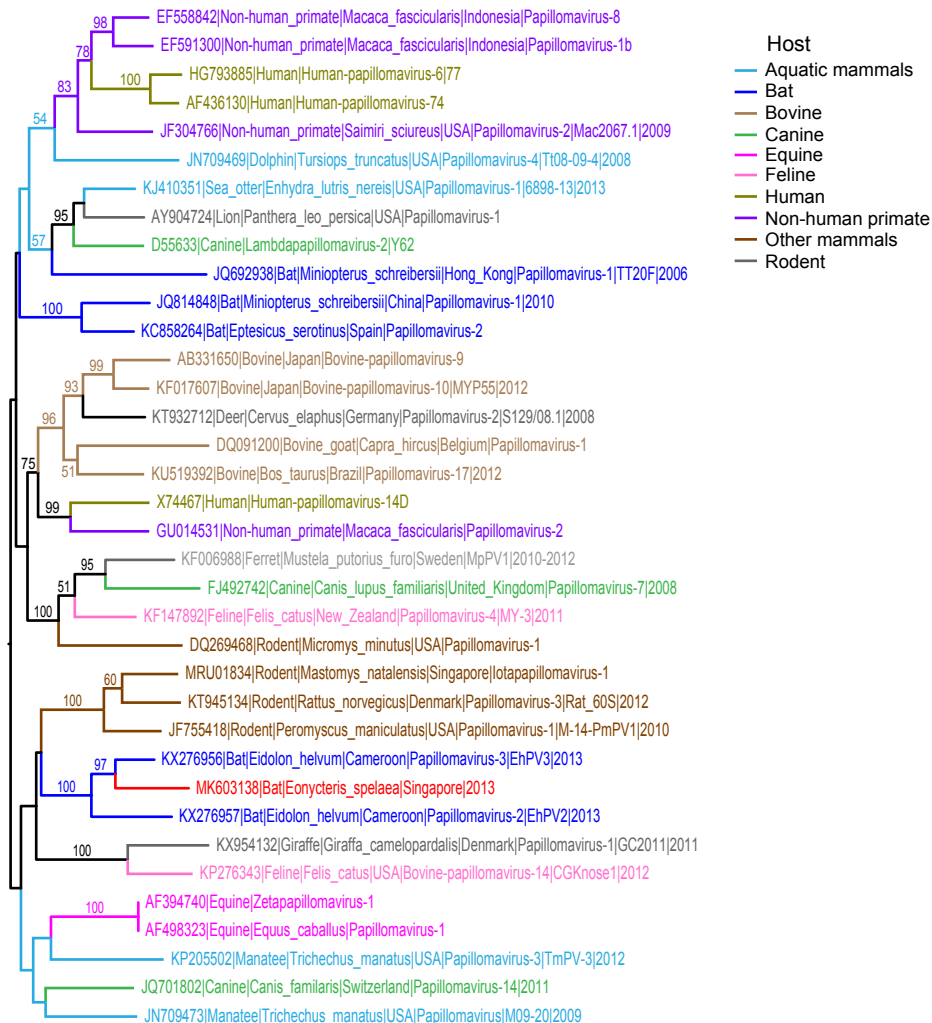

Supplement: Supplementary file 1 [file viruses-11-00250-s001.zip › 2-viruses-447243-suppl/SFig_03_Papilloma.pdf]

# Polyomavirus (VP2)

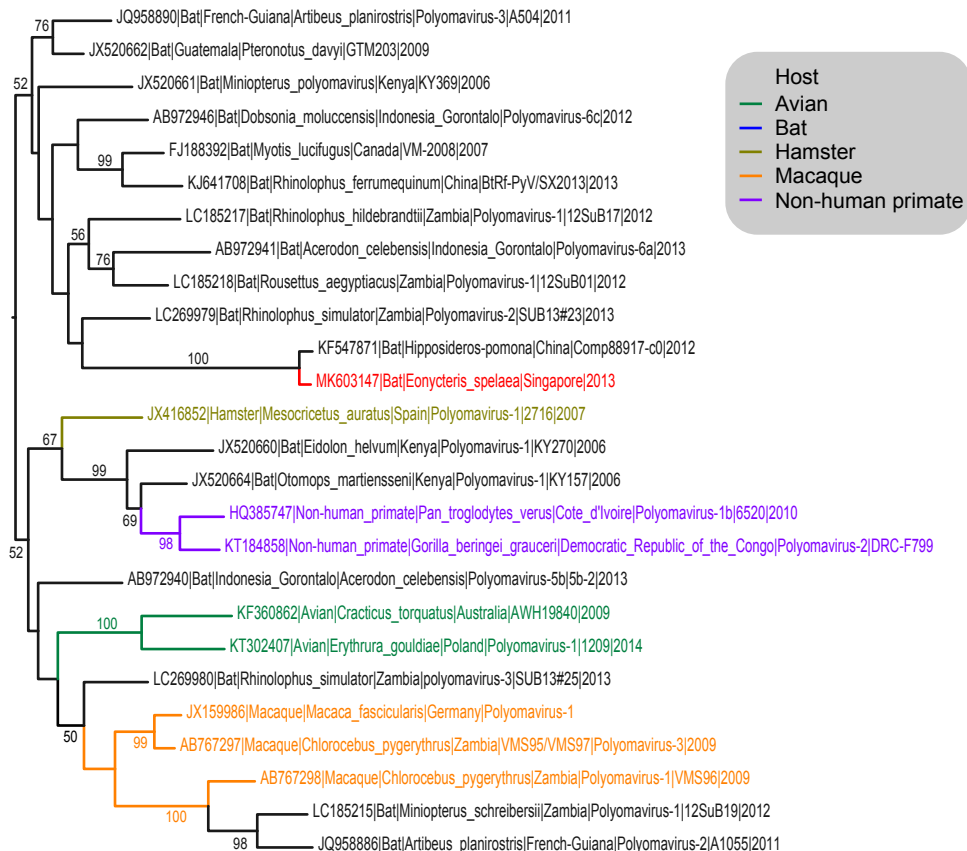

0.2

Supplement: Supplementary file 1 [file viruses-11-00250-s001.zip › 2-viruses-447243-suppl/SFig_05_Polyomavirus.pdf]
